# Research on Creep Behaviors of GH3230 Superalloy Sheets with Side Notches

**DOI:** 10.3390/ma18245509

**Published:** 2025-12-08

**Authors:** Honghua Zhao, Dingnan Cheng, Minmin Chen, Wei Xiao, Cheng Hou

**Affiliations:** 1 AECC Hunan Aviation Powerplant Research Institute, Zhuzhou 412002, China; 2 National Key Laboratory of Science and Technology on Aero-Engine Aero-Thermodynamics, Research Institute of Aero-Engine, Beihang University, Beijing 100191, China; 3 School of Mechanical Engineering, Xi’an Jiaotong University, Xi’an 710049, China

**Keywords:** GH3230 superalloy, creep strain curve, creep life, θ parameter method, notch strengthening

## Abstract

**Highlights:**

**What are the main findings?**

- A series of creep tests on GH3230 superalloy smooth and notch specimens were conducted.
- The creep strain curves and creep life were predicted by the θ parameter method
- Notches exhibited a creep life enhancing effect on GH3230 superalloy under the same net stress level.

**What are the implications of the main findings?**

- The notched specimens exhibited a significantly accelerated tertiary creep stage.
- The creep strain curves and creep life predicted by the θ parameter method were in good agreement with the test results.
- Both stress concentration factor and the net stress collectively determined the notch life enhancement factor.

**Abstract:**

In order to study the effects of notches on the creep behaviors of GH3230 superalloy, a series of creep tests were conducted on GH3230 superalloy sheets with edge notches at 900 °C and 1000 °C. The creep strain curves and creep life of smooth flat plate specimens were predicted based on the θ parameter method. The results indicated that the second stage of steady-state creep of smooth flat plate specimens dominated the creep process, while the strain rate of notched specimens increased significantly in the third stage. The creep fracture strain gradually decreased with increasing creep load. The creep strain curves obtained based on the θ parameter method were in good agreement with the experimental creep strain curves. The predicted creep life of the smooth flat plate specimens and notch specimens were in good agreement with the experimental results, and all experimental results were within the double dispersion band of the predicted life. Notches exhibited a creep life enhancing effect on GH3230 superalloy under the same net stress level. Both stress concentration factor and the experimental net stress collectively determined the notch life enhancement factor. The higher the experimental net stress, the more pronounced the notch life enhancement effect.

## 1. Introduction

GH3230 nickel-based superalloy is a new generation of Ni-Cr-based solid solution strengthened deformation high-temperature alloy developed in China. Due to its simple chemical composition, high strength, weldability, and oxidation resistance, it is mainly used in the combustion liners of aircraft engines [1,2]. Despite these advantages, prolonged exposure to harsh high-temperature environments inevitably leads to microstructural degradation and performance deterioration in GH3230, thereby reducing engine reliability and directly compromising the operational lifespan and flight safety of aero-engines [3,4].

Among the factors influencing combustion liner longevity, creep deformation failure is a predominant failure mode for hot-section components in aero-engines, critically determining the service life of combustion liners [5,6,7]. Moon et al. [8,9] identified steady-state thermal load-induced creep damage as the primary cause of combustor failure. By applying thermal loads as boundary conditions and employing the finite element method (FEM), the thermal stress distribution in combustor substrates was calculated. Using the Larson–Miller parameter-based fracture curve of the substrate material, the creep life of combustion liners was predicted, achieving strong consistency between predicted results and actual operational service durations. Yang et al. [10] studied the creep rupture behavior and notch effect of DD6 Ni-based single-crystal superalloy through creep tests conducted in atmospheric air at 950 °C. The experimental results indicate that the notched specimen has a significant strengthening effect. The competitive mechanism between maximum principal stress and stress triaxiality leads to creep notch strengthening effect, where stress promotes fracture but stress triaxiality plays a restraining role. Huang et al. [11] experimentally investigated the V-notch strengthening effect of single-crystal nickel based high-temperature alloy DD6 under continuous and sequential creep loads at 980 ° C. Results showed that the stress fracture life of V-notch specimens was significantly increased. The triaxial stress state caused by the geometric shape of the V-notch limited local deformation, suppressed crack propagation, and prolonged the durability of the material. Guo et al. [12] applied a dislocation damage coupled constitutive model of single-crystal high-temperature alloys to reveal the influence of secondary orientation on the creep life of circular holes. Results indicated that geometrically necessary dislocations can enhance dislocation hardening near holes, prolong predicted lifespan, and improve the accuracy of lifespan prediction. Introducing structure-induced geometrically necessary dislocations can help to reduce the conservatism of predicting the creep life of circular holes. Goyal et al. [13,14,15] investigated the notch effects on creep behavior in P91, P92, and Cr-1Mo steels, consistently identifying notch strengthening phenomena. Gong et al. [16] numerically and experimentally analyzed the creep damage and interaction behavior between adjacent notches in creep-resistant components. It was found that although there was interaction between adjacent notches, notches can still extend the creep fracture life. Xu et al. [17] evaluated the effect of notch effect on creep damage and structural strength, and found that the interaction between equivalent stress and stress triaxiality determined the transition between notch strengthening and weakening effects.

Although previous studies have elucidated the conditions and degree of notch-induced creep strengthening, the effect mechanism of the notches on the creep behavior of GH3230 superalloy in high-temperature environments still needs to be studied. Therefore, this study conducted a series of high-temperature creep tests on GH3230 superalloy specimens, including smooth plate specimens and notch specimens with 1 mm and 2 mm diameter notches. A θ parameter method was used to predict the creep strain curves and creep life of smooth specimens. The effect mechanism of notch-induced strengthening on creep life was analyzed in detail. This work provides key guidance for the engineering design and life assessment of combustion liners.

## 2. Materials and Experiments

### 2.1. Materials and Specimens

The GH3230 nickel-based superalloy used in combustion chambers was employed for creep tests. The chemical composition and basic mechanical properties of the GH3230 superalloy are shown in Table 1 and Table 2, respectively [3,18].

**Figure 1:**
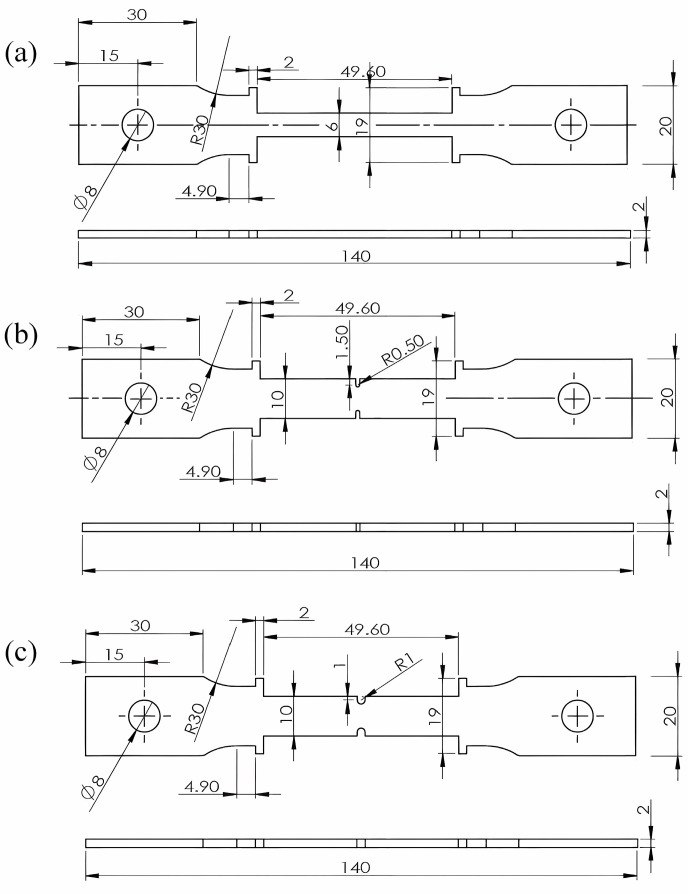
Geometric dimensions of creep specimens. (**a**) Smooth flat plate specimen; (**b**) flat plate specimen with 1 mm diameter notches; (**c**) flat plate specimen with 2 mm diameter notches.

According to standard IS0 204:2009 [19], a series of flat plate creep specimens of GH3230 superalloy were machined with geometric dimensions shown in Figure 1. The creep specimens consist of three types: smooth flat plate specimen, flat plate specimen with 1 mm diameter notches, and flat plate specimen with 2 mm diameter notches. All specimens were ground with 3000 grit sandpaper and mechanically polished to a roughness of Ra = 0.32 μm.

### 2.2. Creep Tests

The number of creep specimens is detailed in Table 3. Smooth flat plate specimens were tested at 900 °C and 1000 °C, while notched specimens with 1 mm and 2 mm diameter circular notches were subjected to creep testing at 900 °C. The net stress refers to the average stress on the cross-section with the minimum area at the notches. To ensure statistical reliability, three replicate tests were conducted for each testing condition.

**Figure 2:**
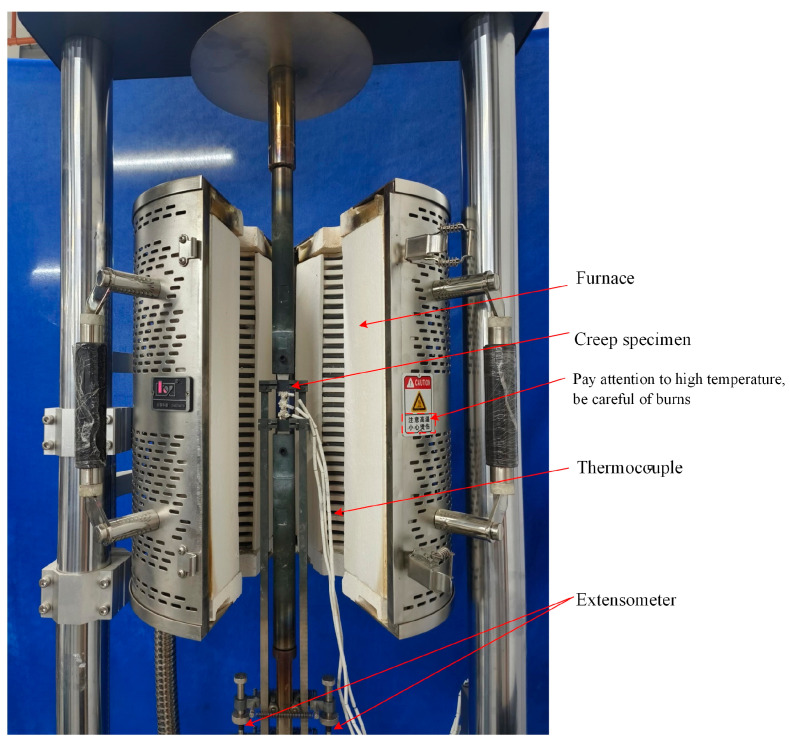
Creep specimen clamping method.

The creep tests were conducted on the creep testing machine RD-100 (Changchun Kexin Experimental Instrument Co., Ltd., Changchun, China), as shown in Figure 2. The test steps were as follows: First, each specimen was fixed on the creep testing machine; subsequently, the high-temperature extensometers and thermocouples were installed and connected; then, the temperature was increased to the target temperature under a constant tensile load of 200 N and maintained for 30 min before the creep tests; at last, the creep tests were conducted. After the specimen creep-fractured, the furnace temperature was cooled to room temperature, and the specimen was removed for subsequent analysis. After creep tests, the creep specimen fracture surfaces were immersed in dilute hydrochloric acid for half an hour, followed by sequential ultrasonic cleaning in acetone and alcohol solutions. After drying, the creep specimen fracture surfaces were observed using a scanning electron microscope (SEM).

## 3. Results and Discussion

### 3.1. Creep Test Results

**Figure 3:**
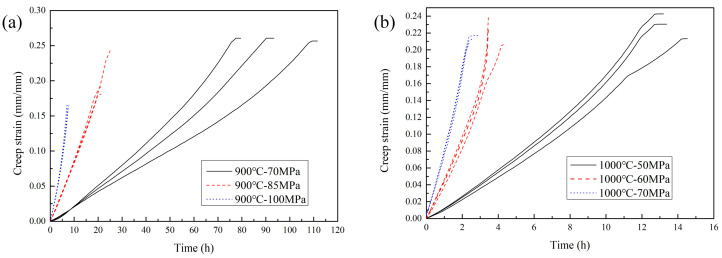
Strain curve test results of smooth flat plate specimen. (**a**) 900 °C; (**b**) 1000 °C.

The test results of the creep strain curves for smooth flat specimens at 900 °C. The test results are shown in Figure 3. The results reveal that the primary creep stage (Creep stage I) of GH3230 superalloy was not obvious, indicating that under the test conditions, the rapid dislocation rearrangement or early onset of dynamic recovery mechanisms in GH3230 was initiated. Steady-state creep (Creep stage II) dominated the deformation process, with a nearly constant minimum creep strain rate. The tertiary creep stage (Creep stage III) was characterized by a rapid acceleration in strain until rupture. At both 900 °C and 1000 °C, the creep fracture strain gradually decreased with increasing creep load. During low-stress load creep conditions, a significant increase in test result dispersion was evident. These findings aligned with typical creep behaviors of high-temperature alloys, where stress-dependent microstructural evolution governed the transition between creep stages.

**Figure 4:**
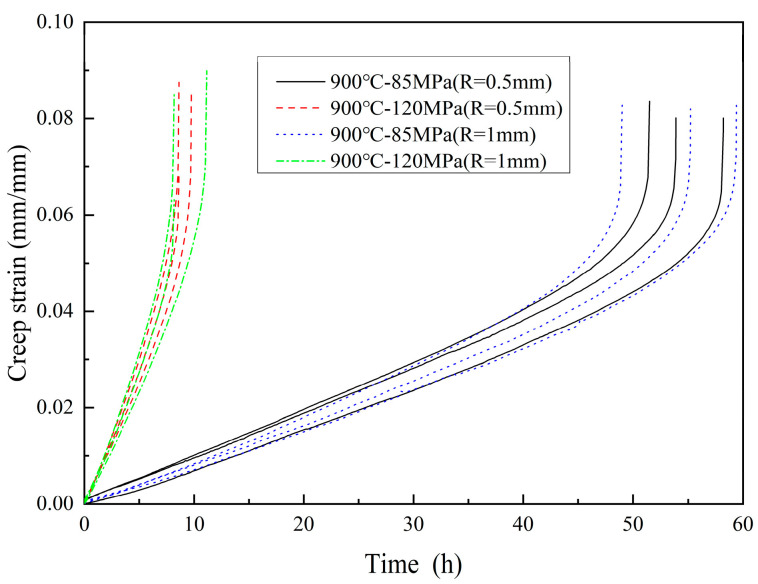
Creep strain curve test results of flat plate specimen with notches at 900 °C.

The creep strain curves of notched flat plate specimens tested at 900 °C are presented in Figure 4. The results revealed notable scatter in the creep curves of notched specimens compared to smooth specimens. For example, of the condition of 900 °C–120 MPa, the creep process of notched specimens exhibited distinct three-stage characteristics. In the primary creep stage, the load was applied instantaneously, and high elastic stress was generated at the root of the notch due to stress concentration, leading to rapid initial plastic deformation. Subsequently, entering the steady-state creep stage, the material at the root of the notch underwent plastic flow and stress relaxation, resulting in a redistribution of stress in the area and a decrease in the actual stress level. Meanwhile, the strong three-dimensional stress constraint suppressed the lateral shrinkage of the material, forming a local strengthening zone at the root of the notch. At this stage, the overall deformation is small and the rate is stable. With the further development of creep, entering the accelerated tertiary creep stage, damage accumulated significantly in the root area of the notch, and micro-creep voids nucleated, connected, and formed microcracks here. When the main microcrack initiated from the root of the notch, it rapidly expanded inward, the effective bearing section decreased sharply, and the deformation accelerated abruptly until the specimen finally fractured at the notch section. Additionally, the observed scatter in creep curves may arise from variations in notch-tip stress distribution due to minor geometric inconsistencies or microstructural heterogeneities near the notch root. These findings underscored the critical role of geometric discontinuities in amplifying creep damage mechanisms under high-temperature loading conditions.

**Figure 5:**
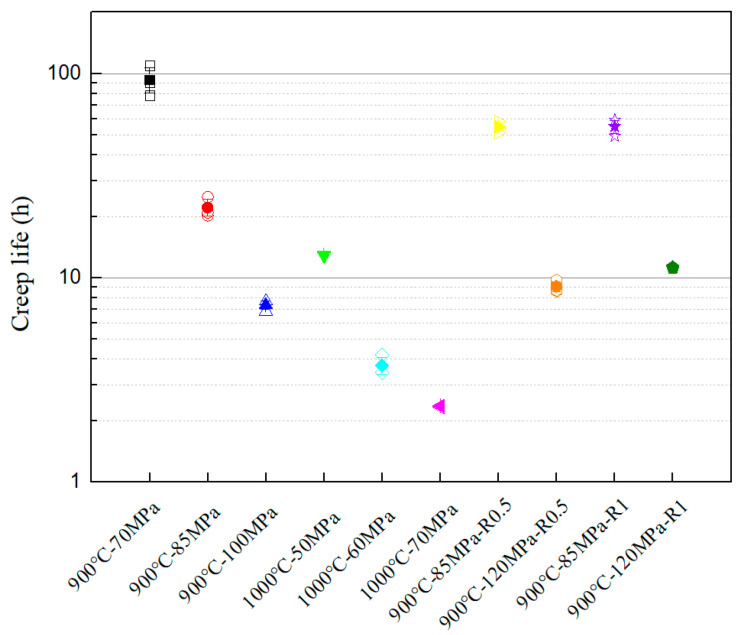
Creep fracture life test results.

The creep fracture life results for all specimens are summarized in Figure 5. The data exhibited low scattering and tight clustering, indicating high experimental reproducibility. At a given temperature, the creep fracture life decreased with increasing applied net stress. Notably, under the same net stress conditions (such as 900 °C–85 MPa condition), the notch specimens had a significantly longer creep fracture life compared to the smooth specimens, demonstrating a significant notch strengthening effect. This enhancement indicated that stress redistribution and local strain adjustment near the root of the notch alleviated damage accumulation, thereby delaying failure. The observed trend was consistent with previous studies on notch-induced creep strengthening in other high-temperature alloys [10,11,12], reinforcing the hypothesis that geometric stress concentrators can paradoxically improve creep resistance under specific thermomechanical conditions. These results emphasized the importance of incorporating notch effects into the life prediction model of combustion liner components.

### 3.2. Stress Analysis of Creep Initial State

**Figure 6:**
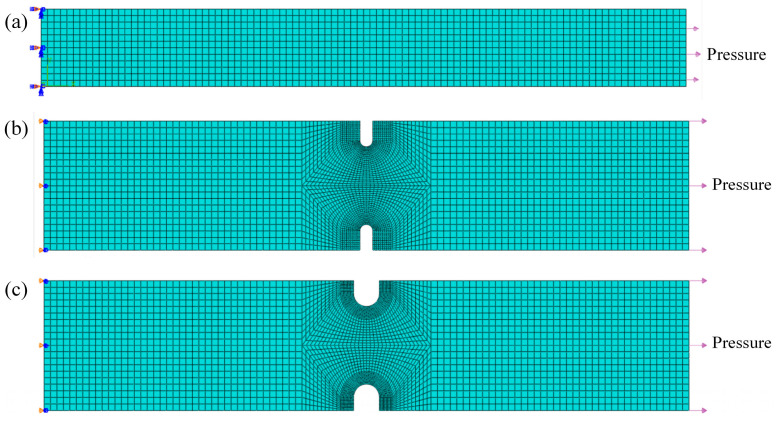
Finite element models of creep specimens. (**a**) Smooth flat plate specimen; (**b**) flat plate specimen with 1 mm diameter notches; (**c**) flat plate specimen with 2 mm diameter notches.

Finite element models of the central parallel gauge sections of smooth and notched flat plate specimens were established using the commercial software Abaqus (Abaqus 6.14), as shown in Figure 6. According to the mechanical boundary conditions, one end of the specimen was fixed, and the other end was subjected to a uniformly distributed pressure load, which was determined based on the creep net stress at the notches. The models were discretized using eight-node linear brick elements with reduced integration (C3D8R). The material parameters of GH3230 superalloy, including elastic modulus and Poisson’s ratio, are shown in Table 2.

**Figure 7:**
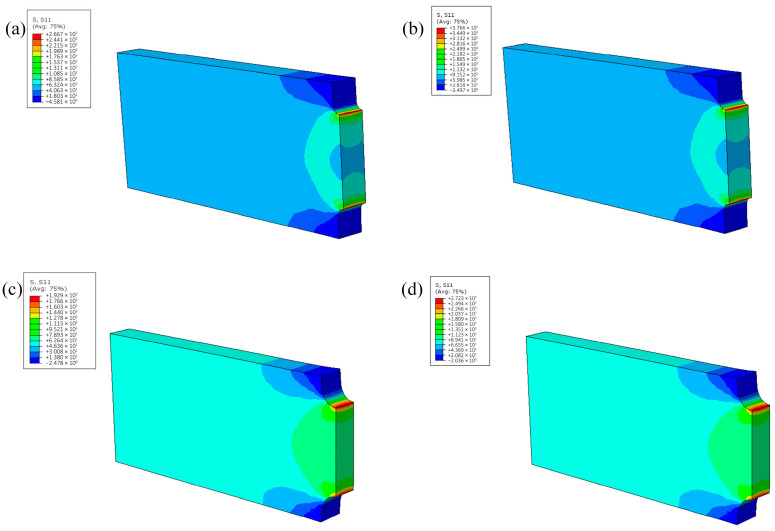
Stress cloud map of creep specimen in tensile direction (S11). (**a**) 900 °C–85 MPa-R0.5; (**b**) 900 °C–120 MPa-R0.5; (**c**) 900 °C–85 MPa-R1; (**d**) 900 °C–120 MPa-R1.

A stress cloud map of a creep specimen in tensile direction (S11) of the notch creep specimens were obtained from finite element calculation, as shown in Figure 7. It can be seen that the stress distribution in the middle cross-section of the notch specimens was uneven, with a larger load bearing at the notch and a smaller load bearing at the center of the section. Pronounced stress concentration persisted at the notch region, and the severity of stress localization intensified with decreasing notch radius. According to stress analysis, the stress concentration factor for the specimens with a 1 mm diameter notch was Kt = 3.13, and Kt = 2.27 for specimens with a 2 mm diameter notch.

### 3.3. Creep Life Prediction

#### 3.3.1. Prediction of Creep Strain Curves Based on θ Parameter Method

The θ parameter method, initially proposed by Evans et al. in 1986 [20], is to establish a mathematical model between material creep characteristics and service condition parameters (such as temperature, stress, and so on) to achieve accurate prediction of creep life. In this study, a θ parameter method was adopted to characterize the creep strain curves of GH3230 superalloy.

(1) $$\varepsilon =\varepsilon_{0}+\theta_{1}t+\theta_{2}(\exp(\theta_{3}t)-1)$$

Among them, ε represents the total creep strain during the creep process, t is the creep time, and ε0 is the initial strain. The θ coefficients (θ_1_, θ_2_, and θ_3_), related to creep temperature and stress, satisfy the relationship defined in Equation (2).

(2) $$\mathrm{lg}\theta_{i}=a_{i}+b_{i}T+c_{i}\sigma +d_{i}T\sigma$$

In Equation (2), T represents the creep temperature, σ denotes the creep stress. ai, bi, ci, and di (i = 1, 2, or 3) are material-dependent coefficients that can be obtained through multiple linear regression analysis of creep test data. Based on the creep test results of smooth flat plate specimens of GH3230 superalloy, the parameters θ_1_, θ_2_, and θ_3_ for the θ parameter method were fitted and are presented in Table 4.

Additionally, a polynomial linear fitting was applied to the data in Table 4, and the corresponding values of *a_i_*, *b_i_*, *c_i_*, and *d_i_* (*i* = 1, 2, or 3) in Equation (2) can be obtained, as presented in Table 5.

**Figure 8:**
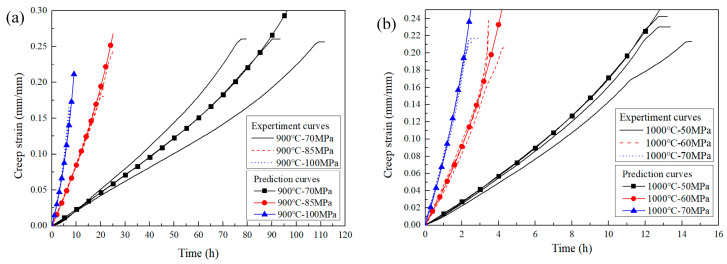
Creep strain prediction curves of smooth flat plate specimens based on θ parameter method. (**a**) 900 °C; (**b**) 1000 °C.

For the GH3230 superalloy material, the creep strain prediction curves obtained using the θ parameter method are shown in Figure 8. It can be observed that the creep strain prediction curves exhibited good agreement with the experimental creep curves. This demonstrated that the θ parameter method can effectively and accurately characterize the uniaxial creep strain behaviors of the GH3230 superalloy.

#### 3.3.2. Creep Life Prediction for Smooth Flat Plate Specimens

By differentiating Equation (1), the creep strain rate equation can be obtained.

(3) $$\dot{\varepsilon }=\theta_{1}+\theta_{2}\theta_{3}\exp(\theta_{3}t)$$

where $\dot{\varepsilon }$ is the creep strain rate. When *t* = 0, the minimum creep strain rate is obtained.

(4) $${\dot{\varepsilon }}_{\min}=\theta_{1}+\theta_{2}\theta_{3}$$

If the creep fracture strain *ε_r_* of a material was known, the creep fracture time, i.e., the creep life *t_f_*, can be obtained from the creep strain curves. Dyson et al. [21] summarized the law of creep fracture strain*ε_r_* through extensive creep tests.

(5) $${\dot{\varepsilon }}_{\min}\cdot t_{r}=1/C$$

(6) $$\varepsilon_{r}\leq \frac{n}{\alpha (n+C)}$$

In which ${\dot{\varepsilon }}_{\min}$ is the minimum creep rate; *t_r_* is the creep fracture time; *C* is a material constant; *n* is the stress exponent; *α* is a parameter related to the test material, and *α* = 1.9 was adopted in this study. Therefore, the minimum creep rate ${\dot{\varepsilon }}_{\min}$ and creep rupture time *t_r_* can be obtained from creep test results. Subsequently, the material constant C can be determined. The stress exponent *n* can then be calculated using the Norton equation ${\dot{\varepsilon }}_{\min}=B\sigma^{n}$. Finally, the creep fracture strain *ε_r_* of the material can be derived from Equation (6), and the creep life *t_f_* can be predicted based on the creep strain prediction curves obtained from the *θ* parameter method. Based on the experimental data from smooth flat plate specimens of GH3230 superalloy, the predicted creep life *t_f_* of GH3230 superalloy is summarized in Table 6.

**Figure 9:**
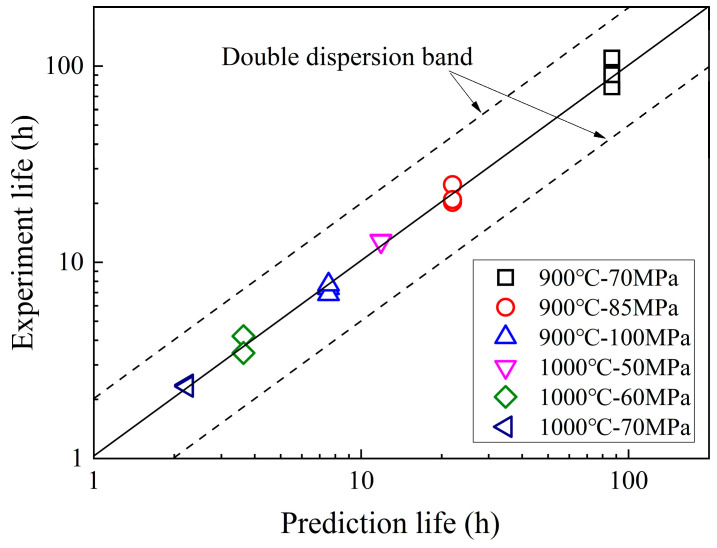
Comparison of predicted and experimental life of smooth flat plate specimens of GH3230 superalloy.

The comparison between the predicted and experimental creep life of GH3230 superalloy smooth flat plate specimens is shown in Figure 9. It can be observed that the creep life prediction results based on the *θ* parameter method were in good agreement with experimental results, and all experimental results were within the double dispersion band of the predicted life.

#### 3.3.3. Creep Life Prediction for Notched Specimens

Under the condition of 900 °C, the parameter C in Equation (5) and the creep stress σ were fitted using a power-law function.

(7) $$C=a\sigma^{b}$$

The coefficients are determined as *a* = 1.924 × 10^−4^ and *b* = 2.384. Consequently, the creep life of smooth flat plate specimens under the condition of 900 °C can be predicted using Equations (1)–(6). Thus, the notch life enhancement factor, which is defined as the ratio of the notched specimen creep life to smooth flat plate specimen creep life under the same net stress condition, can be obtained as shown in Table 7. This study employed the notch life enhancement factor to quantify the strengthening effect of the notch on creep life. It can be observed that the notch enhanced the creep life under the same net stress level. However, the stress concentration factor at the edge of the hole for different notch radii had a relatively small impact on the creep life. A larger notch life enhancement factor indicated a more significant notch strengthening effect. The notch life enhancement factor was closely related to the applied load magnitude and the stress concentration near the notch.

**Figure 10:**
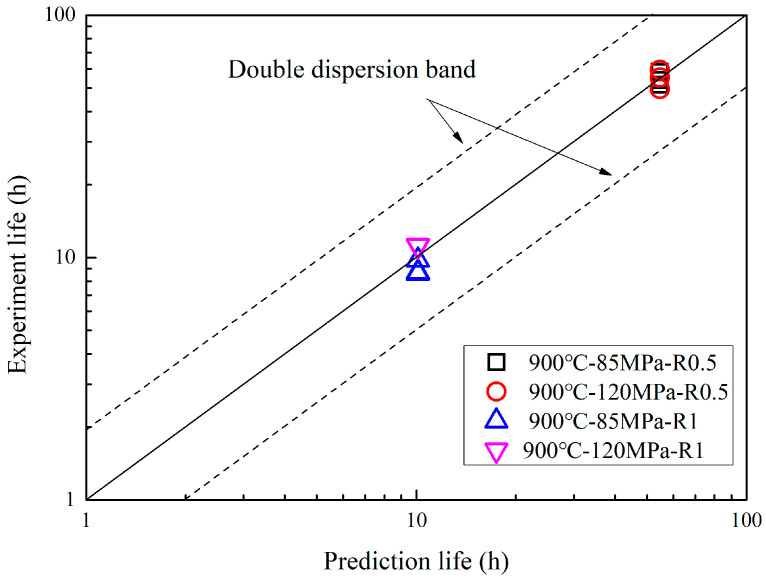
Comparison of predicted and experimental life of notch specimens of GH3230 superalloy at 900 °C.

Therefore, based on the creep life prediction results from smooth flat plate specimens, the creep life prediction for notched specimens can be obtained by a notch life enhancement factor. Figure 10 showed the comparison of predicted and experimental life of notch specimens of GH3230 superalloy at 900 °C. It can be found that the creep life prediction results were in good agreement with experimental results, and all experimental results were within the double dispersion band of the predicted life.

**Figure 11:**
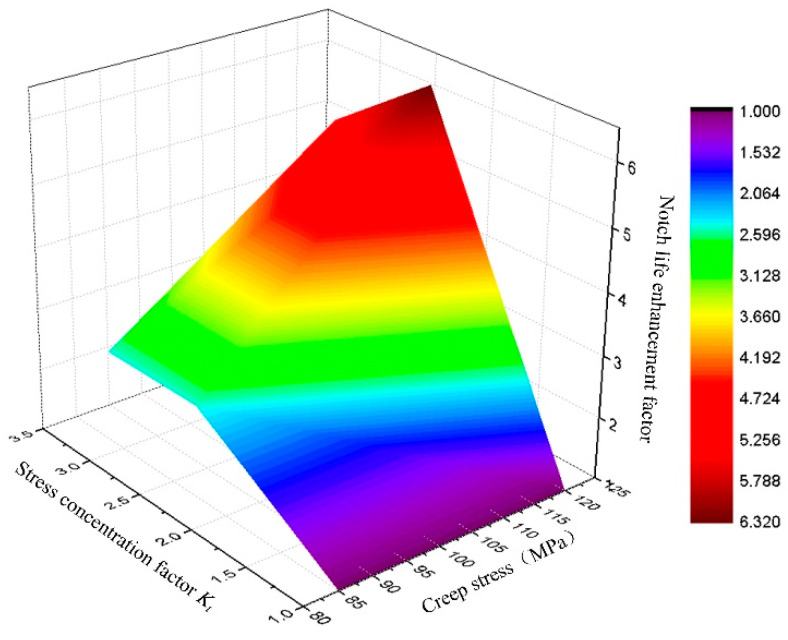
Cloud map of notch life enhancement factor for GH3230 superalloy at 900 °C.

The contour map of the notch life enhancement factor for the GH3230 superalloy at 900 °C was shown in Figure 11. It can be observed that both stress concentration factor and the experimental net stress collectively determined the notch life enhancement factor. The higher the experimental net stress, the more pronounced the notch life enhancement effect.

### 3.4. Fracture Analysis

**Figure 12:**
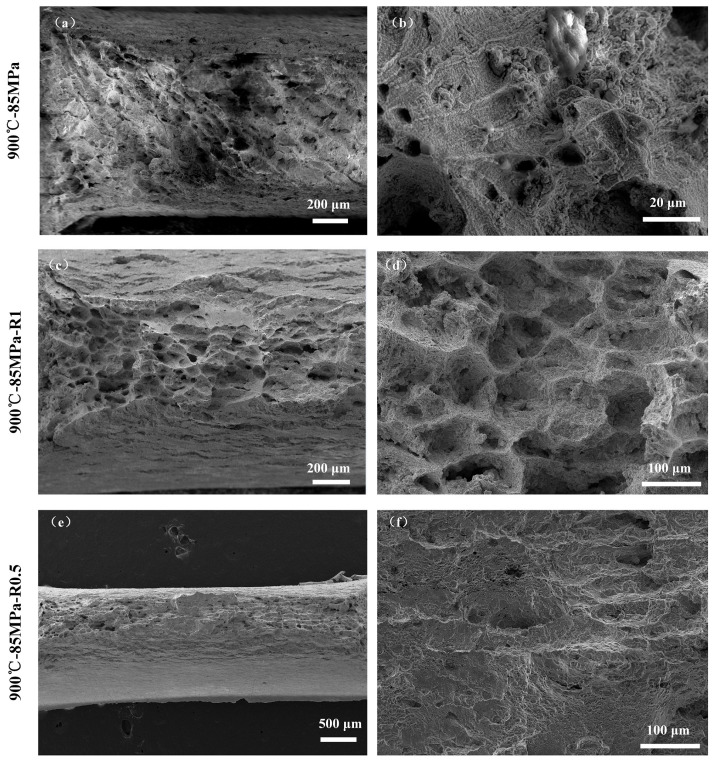
Fracture microstructures of smooth flat plate specimens and notch specimens with 1 mm and 2 mm diameter notches under 85 MPa at 900 °C. (**a**,**b**) Smooth flat plate specimen at 900 °C–85 MPa; (**c**,**d**) notch specimen at 900 °C–85 MPa-R1; (**e**,**f**) notch specimen at 900 °C–85 MPa-R0.5.

The creep specimen fracture surfaces were observed in detail using SEM. The microstructures of the fracture surfaces for smooth flat plate specimens and notch specimens containing 1 mm and 2 mm diameter notches under the conditions of 900 °C–85 MPa are shown in Figure 12. As observed, Figure 12a,b demonstrated that the smooth flat plate specimen fracture surface exhibited numerous dimples and tear ridges, indicating a mixed-mode fracture type dominated by ductile fracture. The fracture was attributed to plastic deformation governed by grain boundary migration. Figure 12c,d revealed that the fracture surface of the specimen with a 2 mm diameter notch contained numerous distinct voids and dimples, characterized by high density and relatively large dimple sizes. Figure 12e,f showed that the fracture surface of the specimen with a 1 mm diameter notch displayed tear ridges and irregular morphologies formed by intergranular crack propagation, presenting a striated fracture surface texture.

## 4. Conclusions

A series of creep tests were performed on smooth and notched (with 1 mm or 2 mm diameter notches) GH3230 superalloy specimens. Based on the θ parameter method, the creep strain curves and creep life of smooth flat plate specimens were predicted, and the notch strengthening effect of the notches on the creep life of GH3230 superalloy was revealed. The conclusions are as follows:

(1) The creep process in smooth flat plate specimens was dominated by the secondary creep stage, whereas the notched specimens exhibited a significantly accelerated tertiary creep stage. The creep fracture strain decreased with increasing load.
(2) Predictions from the θ parameter method agreed well with the experimental creep strain curves and the creep life for both smooth and notched specimens. All experimental results fell within the double dispersion band of the predictions.
(3) Notches exhibited a creep life enhancing effect on GH3230 superalloy under the same net stress level. The degree of this notch life enhancement was governed by both the stress concentration factor and the applied net stress, with the effect being more pronounced at higher stress levels.

## Figures and Tables

**Table 1:** Chemical composition of the GH3230 superalloy (wt.%) [3,18].

Elements	C	Cr	Ni	Co	W	Mo	Al	Ti
Mass fraction/%	0.05–0.15	20.00–24.00	balance	≤5.00	13.00–15.00	1.00–3.00	0.20–0.50	≤0.10
Elements	Fe	La	B	Si	Mn	S	P	Co
Mass fraction/%	≤3.00	0.005–0.05	≤0.015	0.25–0.75	0.30–1.00	≤0.015	≤0.05	≤0.50

**Table 2:** Basic mechanical properties of the GH3230 superalloy [3,18].

Temperature/°C	Elasticity Modulus *E*/GPa	Poisson Ratio *v*	Yield Strength/MPa	Tensile Strength/MPa	Thermal Expansion Coefficient *α*/10^−6^ °C^−1^
25	215	0.31	389.5	905~910	/
600	182	0.32	316.6	735~740	14.7
700	176	0.33	289.9	620~635	15.3
800	168	0.33	234.2	405~410	15.7
900	160	0.34	142.7	250~285	16.0
1000	150	0.35	71.5	151~157	16.3

**Table 3:** Creep test conditions of GH3230 superalloy.

Temperature/°C	Notch	Net Stress/MPa	Number of Specimens
900	Smooth flat plate specimen	100	3
		85	3
		70	3
1000	Smooth flat plate specimen	70	3
		60	3
		50	3
900	R = 0.5 mm	120	3
		85	3
	R = 1 mm	120	3
		85	3
Total			30

Note: Net stress is the average stress at the parallel segment for smooth flat plate specimen or the notch for notch specimen.

**Table 4:** The fitting values of *θ*_1_, *θ*_2_, and *θ*_3_ obtained by the *θ* parameter method.

Temperature/°C	Stress/MPa	*θ*_1_	*θ*_2_	*θ*_3_
900	70	0.0023	0.0012	0.0443
	85	0.0068	0.0126	0.0873
	100	0.0093	0.0217	0.2149
1000	50	0.0113	0.0107	0.1872
	60	0.0281	0.0245	0.4448
	70	0.0440	0.0370	0.6300

**Table 5:** The fitting values of *a_i_*, *b_i_*, *c_i_*, and *d_i_* (*i* = 1, 2, or 3) in Equation (2).

*θ*	*a_i_*	*b_i_*	*c_i_*	*d_i_*
*θ*_1_	−10.729	0.00574	−0.09642	0.00010
*θ*_2_	−33.419	0.02363	0.20371	−0.00014
*θ*_3_	−4.5575	0.00164	−0.16337	0.00016

**Table 6:** Predicted creep life of GH3230 superalloy according to the *θ* parameter method.

Temperature/°C	Net Stress/MPa	${\dot{\varepsilon }}_{\min}$/h^−1^	*t_r_*/h	*C*	*n*	*ε_r_*	*t_f_*/h
900	70	0.0023	92.8	4.66	4.21	0.25	86.6
	85	0.0079	22.0	5.79	4.21	0.22	22.0
	100	0.0139	7.3	9.83	4.21	0.16	7.6
1000	50	0.0133	12.9	5.87	4.25	0.22	11.9
	60	0.0390	3.7	6.92	4.25	0.20	3.6
	70	0.0673	2.4	6.32	4.25	0.21	2.2

Note: Net stress is the average stress at the parallel segment for the smooth flat plate specimen or the notch for notch specimen.

**Table 7:** Notch life enhancement factors for notched specimens at 900 °C.

Stress/MPa	Notch Radius/mm	Life/h	Stress Concentration Factor *K_t_*	Notch Life Enhancement Factor	
85	R = 1	54.71	2.27	2.489	2.485
85	R = 0.5	54.53	3.13	2.481	
120	R = 1	11.20	2.27	6.32	5.71
120	R = 0.5	9.03	3.13	5.1	

## Data Availability

The original contributions presented in this study are included in the article. Further inquiries can be directed to the corresponding author.
